# Hysteroscopy combined with laparoscopy in the diagnosis and treatment of omentum majus incarceration secondary to uterine perforation: A case report and literature review

**DOI:** 10.1111/jog.16213

**Published:** 2025-01-15

**Authors:** Xiaolin Li, Jiandong Hu, Jing Li, Zhonge Tao, Quanxin Qu, Fenge Li

**Affiliations:** ^1^ Department of Gynecology Tianjin First Central Hospital Tianjin China; ^2^ Core Laboratory Tianjin Beichen Hospital of Nankai University Tianjin China; ^3^ Department of Oncology Tianjin Beichen Hospital of Nankai University Tianjin China

**Keywords:** combined hysteroscopic and laparoscopic, complication, induced abortion, omentum incarceration, uterine perforation

## Abstract

Cervical dilatation, uterine evacuation, and curettage (D&E&C) are common gynecological procedures for abortion, yet they carry risks of complications such as uterine perforation and intra‐abdominal organ incarceration. Here, we report a rare case of a breastfeeding patient who had an embedded abdominal greater omentum in the anterior wall of the uterus and into the uterine cavity during D&E&C. We used combined hysteroscopic and laparoscopic treatment for this case and successfully removed the embedded greater omentum. Our experience underscores the importance of vigilant intraoperative monitoring and prompt management to prevent serious complications like infection and bowel injury. In conclusion, hysteroscopic and laparoscopic combination treatment can be a preferred approach to avoid serious adverse outcomes for uterus perforation patients who developed omentum majus incarceration.

## INTRODUCTION

Maternal mortality remains a significant global health concern, often exacerbated by complications from induced abortion.^1^, ^2^ Approximately 14% of maternal deaths are attributed to complications related to induced abortions, including hemorrhage, sepsis, and uterine perforation.^1^, ^3^ Developing countries bear the brunt of these risks, with unsafe abortions contributing substantially to mortality rates.^4^ In this report, we describe an uncommon case of asymptomatic incarceration of the greater omentum within the uterus, which occurred as a consequence of uterine perforation resulting from curettage during an induced abortion. The diagnosis was established 2 months following the abortion procedure. We employed a combined approach utilizing both hysteroscopic and laparoscopic techniques to successfully excise the incarcerated greater omentum and repair the uterine wall. This case underscores the potential efficacy of combined hysteroscopic and laparoscopic interventions for addressing greater omentum incarceration in the uterus. Furthermore, it serves as a cautionary note for gynecologists to enhance their clinical proficiency in managing induced abortions and to minimize complications associated with such procedures.

### Case presentation

**FIGURE 1:**
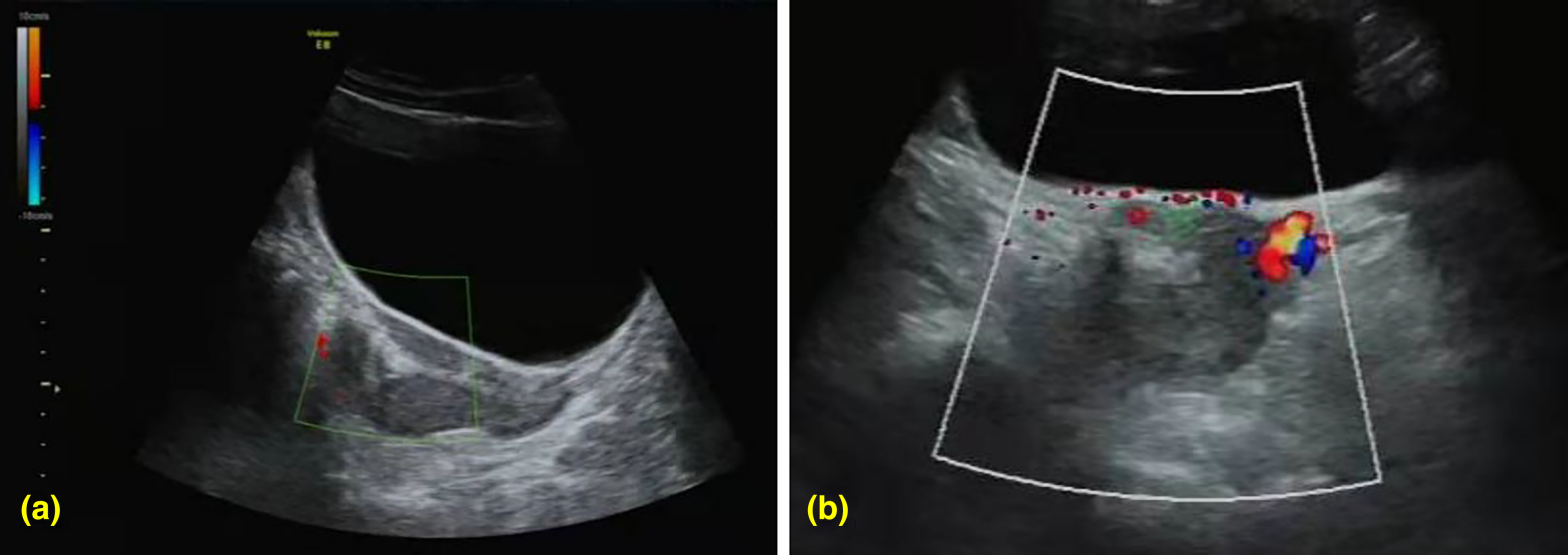
Ultrasound image shows the presence of a hyperechogenic structure in the anterior wall of the uterus (a, b).

**FIGURE 2:**
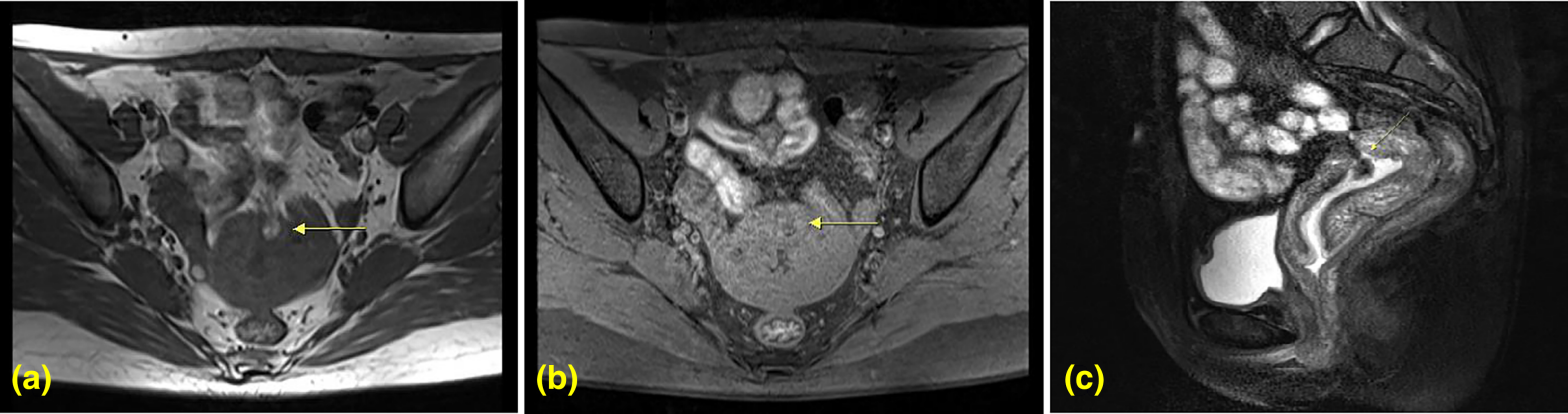
Pelvic magnetic resonance imaging (MRI) showed that adipose tissue was embedded in the anterior wall of the uterus to the uterine cavity by axial T2‐weighted image (a), axial fat‐suppressed T1‐weighted image (b), and sagittal fat‐suppressed T2‐weighted image (c).

A 28‐year‐old patient breastfeeding presented 2 months after undergoing D&E&C at 10 weeks of gestation. During the procedure, a rigid vacuum aspiration cannula was employed, which unexpectedly became immobile, raising concerns regarding potential uterine perforation. Although the patient did not exhibit immediate symptoms, she postponed her follow‐up appointment scheduled for 1 week later. Upon ultrasound examination 1 month later, findings indicated incomplete uterine serosa and a hyperechoic structure located in the anterior uterine wall (Figure 1a, b), which suggested the possibility of incarceration of the abdominal omentum majus. Magnetic resonance imaging (MRI) subsequently confirmed this diagnosis, revealing fatty tissue consistent with omentum majus within the uterine cavity (Figure 2a–c). A physical examination showed no signs of abdominal distension or tenderness, and laboratory analyses indicated a serum ß‐hCG level of 1.38 IU/mL, thereby excluding the presence of residual embryonic tissue. Written informed consents were obtained from the patient to participate in the study. Written informed consent was obtained from the patient for publication of the details of their medical case and any accompanying images.

A physical examination of the abdomen revealed no signs of swelling or tenderness. The pelvic examination also yielded no significant findings. Laboratory tests, including a complete blood count, prothrombin time, activated partial thromboplastin time, serum electrolytes, serum urea, and serum creatinine, all returned normal results. The incarcerated tissue was ruled out as the intestinal tube based on findings from both ultrasound and MRI. Based on the diagnosis, we decided to proceed with a combined hysteroscopic and laparoscopic procedure.

**FIGURE 3:**
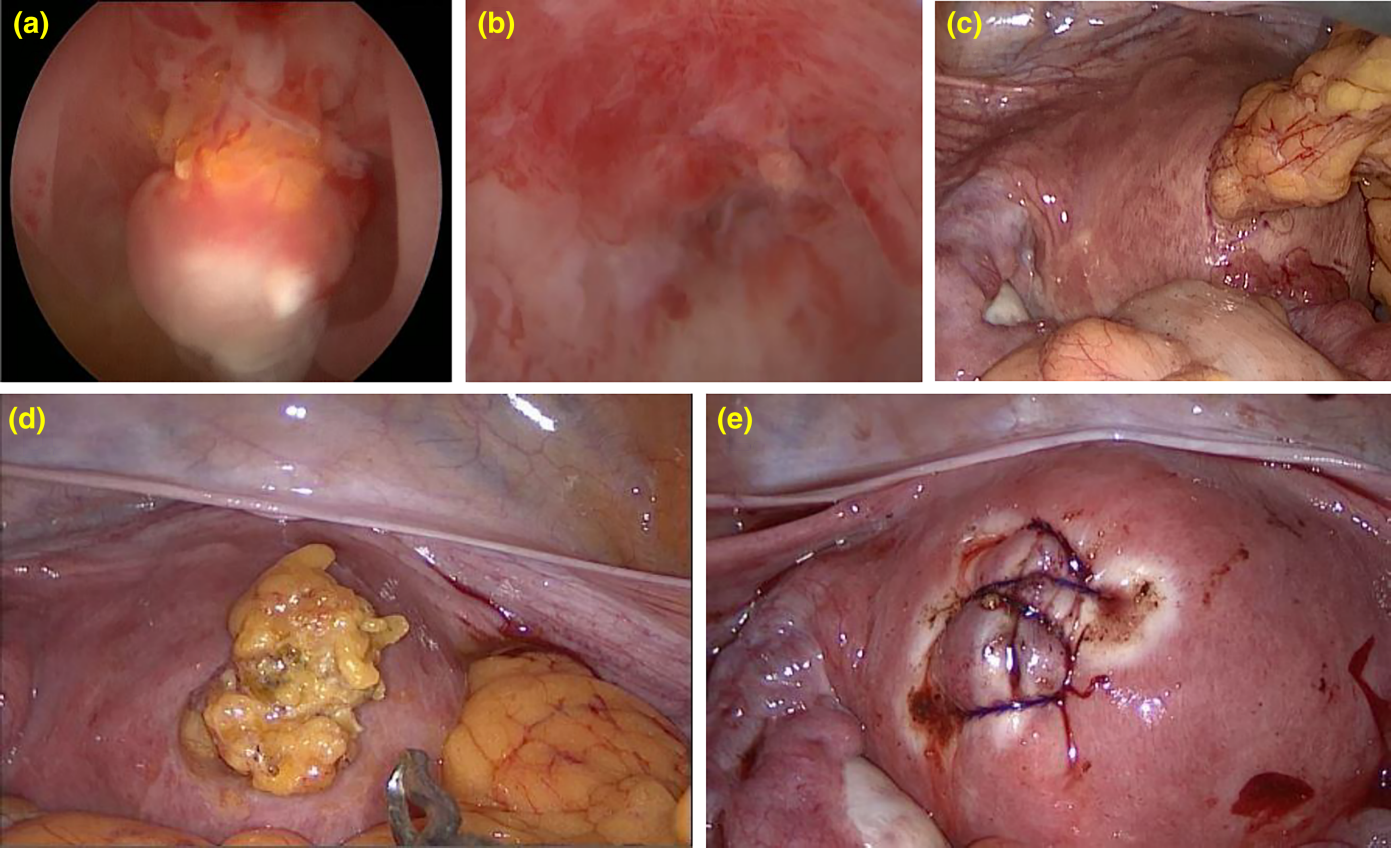
Hysteroscopy examination revealed a pale‐pink mass with intrauterine (a). Final hysteroscopic. view with the omentum completely released (b). Laparoscopy showed an incarcerated omentum into uterine cavity through the uterine perforation site on anterior uterine wall (c). Cut off the omentum incarcerated (d). After the suture of the anterior uterine wall defect (e).

Hysteroscopy revealed a pale‐pink mass protruding into the uterine cavity (Figure 3a, b), confirmed as the incarcerated omentum majus. Laparoscopy corroborated this finding, identifying the omentum majus through a perforation in the anterior uterine wall (Figure 3d, e). We conducted a laparoscopic removal of the omentum, which was followed by stitching up a defect in the uterine wall. During the operation, fluid from the uterus leaked into the abdominal cavity, leading to a temporary halt in hysteroscopic monitoring until the uterine wall was closed laparoscopically. After the procedure, a hysteroscopy verified that there was no remaining omentum majus in the uterine cavity. The patient had a smooth recovery and was discharged 2 days post‐surgery. This study received approval from the ethical committee of Tianjin First Central Hospital, and informed consent for publication was obtained from the patient.

## DISCUSSION

Unsafe abortions pose a significant public health challenge, with an estimated 25.1 million unsafe procedures occurring annually worldwide.^5^ Risk factors for uterine perforation include uterine anomalies, prior scar tissue, and inadequate cervical preparation, particularly when performed by inexperienced operators.^3^ The predominant factor contributing to uterine perforation is frequently the erroneous assessment of the uterus's anatomical position. This misjudgment may result in the posterior aspect of the uterus being incorrectly identified as the anterior, thereby causing the vacuum aspiration cannula, when subjected to negative pressure, to perforate the anterior wall of the uterus. Consequently, this can lead to the omentum majus being inadvertently drawn into the uterine cavity.

During gestation, the uterus exhibits a notable increase in softness, which heightens the likelihood of uterine perforation in patients who are lactating. The vascular architecture of the uterus is defined by the uterine artery, which ascends laterally and bifurcates into arcuate arteries that progressively infiltrate the myometrium, with a relatively sparse distribution of robust blood vessels in the central region. Consequently, perforations are predominantly observed in the mid‐uterine area, typically resulting in minimal hemorrhage, whereas perforations in the lateral wall may lead to the formation of hematomas within the broad ligament. In instances where the perforation is small and not subjected to persistent mechanical stress, the affected area tends to contract rapidly. Furthermore, in cases of inadvertent perforation, the application of curettage suction may unintentionally aspirate portions of abdominal organs through the uterine wall.^6^

The clinical presentations of uterine perforation can vary significantly, ranging from asymptomatic cases to those exhibiting mild or severe, potentially life‐threatening symptoms. The severity of symptoms is influenced by the location of the perforation and the surgical instruments employed during the procedure. The majority of perforations are located in the body of the uterus, with approximately 40% occurring in the anterior wall, 36% in the cervix, and around 13% in the fundus. Generally, patients remain asymptomatic if the perforation is caused by a probe or a palace strip, provided that it is promptly identified and managed. The clinical symptoms associated with uterine perforation may include: (1) injury to the uterine artery, which can lead to the formation of a broad ligament hematoma; (2) persistent bleeding that may result in abdominal pain, hypotension, and potentially shock; (3) if there is an injury to the bowel, patients may present with fever, abdominal pain, distension, peritonitis, intestinal obstruction, hematuria, or hypotension; and (4) severe hemorrhage or complications arising from sepsis can precipitate shock, which poses a significant risk to life.^3^, ^7^, ^8^

Patients who experience uterine perforation and incarceration of the omentum majus may be asymptomatic, as illustrated in the case mentioned above, or may only suffer from moderate chronic pelvic pain^9^, ^10^ and patients can remain asymptomatic for many years. A rare case^8^ has been documented involving a postmenopausal woman who was diagnosed with omental incarceration 23 years subsequent to a dilation and curettage (D&C) procedure. Furthermore, two additional cases were reported concerning unsafe abortions, in which patients suffered from uterine perforation that resulted in intestinal evisceration through the vaginal canal, ultimately causing acute intestinal obstruction.^3^ These patients often present with severe abdominal pain accompanied by vomiting and difficulties with defecation and gas expulsion.

Multiple scenarios suggest the potential occurrence of uterine perforation: (1) when the surgical instrument ceases to encounter resistance from the uterine wall or exhibits a loss of stability during the procedure; (2) if vacuum aspirators are suddenly and rapidly maneuvered during the operation; (3) when adipose tissue is encountered during curettage; and (4) if patients report severe abdominal pain, substantial hemorrhaging, the emergence of hematuria, or hypotension subsequent to an intrauterine procedure.^11^ Patients who sustain uterine perforation as a result of the application of blunt instruments, such as probes, dilators, or curettes utilized without suction and in the absence of symptoms, may frequently be managed through conservative treatment approaches.^3^ Individuals seeking to preserve their fertility and who exhibit persistent defects in the uterine wall should consider surgical intervention to rectify the defect, thereby reducing the likelihood of uterine perforation. Furthermore, it is important to note that individuals with a prior history of uterine perforation typically face an elevated risk of uterine rupture during subsequent pregnancies.^11^

The diagnostic methodologies for uterine perforation typically include the following: (1) real‐time blood analysis to assess leukocyte and hemoglobin levels; (2) ultrasonography of the uterus, which serves as an effective and straightforward diagnostic tool; and (3) MRI, which is adept at identifying incarcerated omental or bowel tissue due to its superior soft tissue contrast, and is applicable when the patient's performance status is stable.^9^

Immediate surgical intervention for uterine perforation is crucial when patients experience ongoing abdominal pain, bleeding, or intestinal damage. Laparoscopy is generally favored over laparotomy as a surgical method. During laparoscopy, the uterine wall defect can be stitched after the removal of trapped tissue, and hysteroscopy can be used to examine any abnormalities within the uterus. In cases of uterine perforation accompanied by necrosis, hysterectomy has also been documented as a treatment option.^12^ Consequently, the integration of laparoscopy and hysteroscopy is regarded as the optimal strategy in this context. Making well‐informed surgical choices concerning uterine perforation can markedly diminish the likelihood of severe complications. The following are several critical recommendations aimed at preventing uterine perforation: (1) obtain a thorough preoperative medical history from patients, including inquiries about breastfeeding status; (2) surgeons should meticulously evaluate the size and positioning of the uterus, as well as the angle and trajectory of instrument insertion; (3) ensure comprehensive cervical preparation while avoiding overly aggressive techniques, contemplate the use of pharmacological or mechanical methods for cervical dilation, as a soft and dilated cervix facilitates easier access for suction aspirators; (4) employ B‐ultrasound guidance during the procedure when deemed necessary; (5) vigilantly monitor any alterations in the patient's symptoms for indications of potential perforation and conduct real‐time evaluations; (6) be prepared to discontinue the procedure if warranted, assess for perforation and tissue penetration, and determine whether conservative or emergency surgical interventions are necessary to avert serious complications. Importantly, there is a most recent follow‐up information which is that this patient got pregnant 6 months after surgery and had been successfully delivered by cesarean section at her 38 weeks pregnancy.

In summary, the management of complications associated with induced abortions, including uterine perforation and the potential incarceration of the omentum majus, necessitates a multidisciplinary strategy and an increased level of clinical vigilance. Gynecologists should be more vigilant and take preventive measures as follows to prevent omentum majus incarceration: before surgery, a detailed medical history needs to be collected to identify patients who are at high risk of uterine perforation; During surgery, gynecologists should stop the operation immediately whenever the suction device has a pause or moving difficult. Timely identification, swift intervention, and the application of advanced surgical methodologies, such as the integration of hysteroscopic and laparoscopic techniques, are essential for reducing severe complications and enhancing patient outcomes.

## AUTHOR CONTRIBUTIONS

**Xiaolin Li:** Conceptualization; data curation; writing – original draft; writing – review and editing. **Jiandong Hu:** Writing – original draft; writing – review and editing. **Jing Li:** Data curation. **Zhonge Tao:** Data curation. **Quanxin Qu:** Writing – original draft. **Fenge Li:** Conceptualization; project administration; supervision; writing – review and editing.

## CONFLICT OF INTEREST STATEMENT

The authors have no conflicts of interest to declare.

## ETHICS STATEMENT

This trial was study was approved by the ethical review board of the ethic committee of Tianjin First Central Hospital (Approval number: B1.202308).

## Data Availability

Due to this work reported a rare clinical case that most of the data including case history, some laboratory test results and images were fully presented in the text, there is very minimal data can be shared. All the data about this patient was in the electric HIS system of the hospital which cannot be shared via any links. Any detailed information can be provided upon reasonable request by contacting the corresponding author.
